# Implementation of a WeChat-Based Smoking Cessation Program for Chinese Smokers

**DOI:** 10.3390/ijerph182111189

**Published:** 2021-10-25

**Authors:** Ting Luo, Mirandy S. Li, Donna Williams, Jackson Fritz, Stephen Phillippi, Qingzhao Yu, Stephen Kantrow, Liwei Chen, Yongchun Chen, Kaylin Beiter, Tung-Sung Tseng

**Affiliations:** 1 Moores Cancer Center, University of California San Diego, La Jolla, CA 92122, USA; 2 Behavioral and Community Health Sciences, School of Public Health, Louisiana State University Health Sciences Center-New Orleans, New Orleans, LA 70122, USA; mli2@lsuhsc.edu (M.S.L.); dwilli3@lsuhsc.edu (D.W.); jfritz@lsuhsc.edu (J.F.); sphill2@lsuhsc.edu (S.P.); kbeite@lsuhsc.edu (K.B.); ttseng@lsuhsc.edu (T.-S.T.); 3 School of Medicine, Louisiana State University Health Sciences Center-New Orleans, New Orleans, LA 70122, USA; skantr@lsuhsc.edu; 4 Biostatistics, School of Public Health, Louisiana State University Health Sciences Center-New Orleans, New Orleans, LA 70122, USA; qyu@lsuhsc.edu; 5 Department of Epidemiology, Fielding School of Public Health, University of California Los Angeles, Los Angeles, CA 900095, USA; cliwei86@g.ucla.edu; 6 Department of Clinical Nutrition, Henan Provincial People’s Hospital, Zhengzhou University People’s Hospital, Zhengzhou 450003, China; henancyc@163.com

**Keywords:** tobacco, smoking cessation, WeChat, implementation, Chinese smokers

## Abstract

**Introduction:** Although smoking prevalence has recently declined, the smoking prevalence in China remains high. Extensive research has demonstrated ways that social media can assist in quitting smoking. WeChat is the most commonly used social media platform in China but has not been used for smoking cessation. A process evaluation of a novel WeChat-based smoking cessation intervention was conducted to measure its efficacy of content delivery, participant satisfaction, engagement, and likelihood of recommendation to others. **Methods:** A three-week, three-arm, single-blind randomized control trial was established. WeChat was used to recruit 403 participants and to deliver intervention messages and process evaluation surveys to them. Recruitment advertisements were posted on an official WeChat account and were forwarded to WeChat Moments. Intervention messages were delivered once a day during weekdays, using the WeChat broadcasting messages function, with two messages being sent each time. Process evaluation surveys were organized using Wenjuanwang and were delivered via WeChat. Process assessments were conducted every Friday to assess intervention message receipt, satisfaction level, engagement level, and recommendation to others. The receipt of intervention messages was measured by a self-reported question indicating which messages were read each week. Satisfaction was measured by a five-item Likert scale survey. Engagement was measured by a one-item Likert scale survey. Recommendation to others was measured by one self-reported question. **Results:** Participants read an average of 4.76 (out of 10), 5.80 (out of 10), and 4.25 (out of 6) messages at week 1, week 2, and week 3, respectively. The second messages were less likely to be read compared to the first messages (52.3% vs. 61.6%, respectively). Moreover, within each single week, the number of participants who read the intervention messages gradually decreases over time. Picture-based intervention messages tended to be less likely to be read than video-based intervention messages. Total program satisfaction scores ranged between 5 and 25, and the overall scores for satisfaction for each week were 21.55, 22.27, and 22.76, respectively. No significant differences were found in all the satisfaction indicators between groups. More than 60% of participants reported being either highly engaged or somewhat engaged each week. In addition, most participants (93.0% at week 1, 95.8% at week 2, and 96.2% at week 3) reported that they were willing to recommend our program to others. **Discussion:** A WeChat-based smoking cessation intervention for Chinese smokers was implemented and evaluated. For future studies, one should consider sending messages of a higher importance as the first message of a given day. Smokers had a higher rate of reading intervention messages at the beginning part of each week, during which, relatively important messages should be prioritized. One might also consider alternating the topics and formats of the messages for a better engagement of the users in future studies.

## 1. Introduction

### 1.1. Smoking Prevalence in China

Studies have demonstrated that smoking tobacco is related to many diseases and various types of cancers, and is the leading cause of preventable morbidity and mortality worldwide [1,2], resulting in the loss of billions of dollars each year for many countries [3]. It is estimated that approximately 6 million people die from tobacco use each year, with many of these deaths occurring prematurely [4,5]. Thus, the tobacco epidemic is considered to be one of the biggest public health threats that the world has ever faced [6]. China is not only the world’s largest tobacco producer, but also consumes the largest amount of tobacco products [7]. Although smoking prevalence has recently declined, the smoking prevalence in China remains high. According to the World Health Organization, in 2018, 26.6% of people aged 15 and above were smokers in China, including 50.5% (300 million) of males and 2.1% of females (14 million) [8]. For comparison, in 2010, 28.1% of people (52.9% of males and 2.4% of females) aged 15 and above were smokers [9]. Nearly 9.5% of all disability-adjusted life years and 16.4% of total deaths for Chinese adults are related to tobacco use in China each year [10]. Therefore, it is necessary to develop prevention strategies to help smokers to quit smoking.

### 1.2. Smoking Cessation in China

Despite an improvement in the smoking cessation rate over the last few decades in China, smoking cessation rates remain low [9]. Only 16.1% of smokers plan to or are thinking of quitting in the next 12 months, and only 19.8% of smokers have made an attempt to quit [8]. Among smokers who attempted to quit, 90.1% of them did not use any cessation methods, including pharmacotherapy medications and counseling [8]. These low cessation attempts, and successes, constitute an important intervention target for global health. Less than one in five of over 300 million smokers in China have successfully quit smoking.

### 1.3. Smoking and Oral Health Diseases 

Smoking tobacco and oral health diseases are highly associated [11]. It is also known that increased overall health awareness is linked with smoking cessation [12]. This high prevalence of oral disease may be partly attributed to low oral health awareness. Oral health awareness is considered low in China when compared with many other counties, a factor that is evidenced in the low rates of treatment of oral health conditions. In China, about 97% of children with carious teeth at age 6 were left untreated, while for children aged 12, this percentage only decreased to 89% [13]. About 79–92% of carious teeth (including third molars) in middle-aged and elderly people are left untreated [13]. In contrast, in the United States, the percentage of children ages 5–19 years and 20–44 years with untreated dental caries were 18.6% and 31.6%, respectively [14]. Improving oral health education may facilitate discussion of overall health status, including the impact of tobacco on dental health and overall bodily health. Since smoking significantly increases the risks of many diseases, an increase in general health awareness may increase intent to quit smoking. In sum, one can consider promoting oral health awareness as a potential avenue to raise individuals’ consciousness about the impact of tobacco use, and thus aid smoking cessation.

### 1.4. The Use of Transtheoretical Model in a Smoking Cessation Intervention

The transtheoretical model (TTM) has been demonstrated to be effective in guiding smoking cessation interventions [15,16,17]. The TTM describes how health behavior change occurs over time through a nonlinear progression of stages [16]. This model incorporates processes and concepts from 300 leading theories of psychological and behavioral change, and has evolved through studies that have examined the experiences of smokers who quit on their own [16]. One major construct of the TTM is the “stage of change”. For each stage of change, various intervention strategies differ in their effectiveness at moving an individual to the next stage of change. In this study, we adapted the TTM to guide our intervention.

### 1.5. Purpose and Objectives

Social media works as a tool for the delivery of smoking-related information and social support and has shown success in studies conducted in the United States, Canada, the UK, and Hongkong [18,19]. In addition, it often costs less to implement an intervention over social media, relative to traditional intervention methods. People in China cannot access many commonly used social media platforms such as Facebook, WhatsApp, Twitter, and Clubhouse. However, WeChat, a multi-purpose messaging, social media, and mobile payment app, is widely used in China. WeChat was first released in 2011 and became the world’s largest standalone mobile app in 2018. WeChat is considered a “super app”, which means the WeChat is an “app for everything”. WeChat now has over 1.2 billion monthly active users, including 1.4 million monthly active users from the US [20]. For comparison, in 2020, Facebook Messenger and Instagram (two other competitive international messaging services better known in the West) had about 1.2 billion monthly active users each [20]. However, to the best of our knowledge, few articles have been published on using WeChat to recruit participants, deliver interventions, and distribute assessment surveys for a smoking cessation intervention. To fill this gap in knowledge, we developed the concepts for, and the application of, WeChat for smoking cessation intervention based on the TTM. The objective of this study was to explore the implementation-related factors—including message delivery, satisfaction, engagement, and recommendation willingness—of a WeChat-based smoking cessation intervention study, based on the TTM.

## 2. Materials and Methods

### 2.1. Study Design

**Figure 1:**
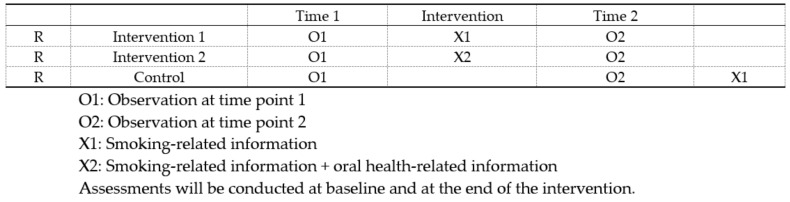
Study design.

This study proposed a randomized controlled trial (RCT) of 330 current smokers. Smokers were randomly distributed between 3 groups, after completing an online baseline assessment. The intervention groups and the control group are outlined in Figure 1. Participants in intervention group 1 received 20 smoking-related messages for 2 weeks. Participants in intervention group 2 received 20 smoking-related messages for 2 weeks and an extra 6 oral health-related messages for an additional third week. Participants in group 3 (waitlist control) received smoking-related messages after the post-intervention assessment. Assessments were conducted at pre-intervention, during the intervention period, and 4 weeks post-intervention. Participants were required to complete a process evaluation survey questionnaire, and this study focused on the results of the process evaluation. All assessment surveys were organized via Wenjuanwang and delivered via WeChat. Wenjuanwang is a popular China marketing research tool, similar to SurveyMonkey [21]. Smoker ID was used to link different surveys. According to our power analysis, this study required a minimal sample size of *n* = 330. All materials and procedures for this study underwent review and approval by the Institutional Review Board (IRB) of the Louisiana State University Health Sciences Center (LSUHSC IRB#: 19-901).

### 2.2. Recruitment

A new WeChat account (ID: QuitSmokingHelp) was created for smoking cessation services, and participants could “friend” this account to show their interest in this study. On 1 July 2019, we posted recruitment advertisements on a WeChat official account (Chinese Clinical Nutrition Network). The recruitment link was as follows: https://mp.weixin.qq.com/s?__biz=MzU5NTY4ODk3Ng==&mid=2247500974&idx=3&sn=36ce00f901181f6c44bbbd9d8c87fa4e&source=41#wechat_redirect (accessed on 1 August 2021). Advertisements for the intervention included images and short text related to the smoking cessation intervention, eligibility criteria, and contact information (WeChat ID, phone number, and the email address of the interventionist). The “word-of-mouth” strategy was applied to recruit participants. We encouraged families, relatives, friends, or any other people who were interested to forward the advertisement to their WeChat Moments, WeChat groups, or any other social media. At the same time, we also encouraged people to post the following recruitment flyers on their WeChat Moments, WeChat groups, or any other social media. A smoker can simply “friend” an interventionist via their QR code, WeChat ID, phone call, or email.

When prospective participants contacted and “friended” the interventionist on WeChat, their eligibility was assessed. They were asked for the last 4 digits of their most frequently used cell phone number, which was used as their “Smoker ID.” We used the last four digits of their most frequently used cell phone number as smoker ID because the last four digits of their phone number are less likely to be forgotten than a random number. The Smoker ID was used to link surveys for pre-intervention, process evaluation surveys, and post-intervention.

Incentives were provided to increase recruitment rate and reduce attrition rate. Participants who completed a baseline assessment or a process evaluation survey (by sending a screenshot indicating the completion of the baseline assessment to the interventionist) received a “Red Packet” with ¥1.88 ($0.30) or ¥1 ($0.15), respectively. The Red Packet feature on WeChat offers users monetary gifts in the form of virtual credit. Red Packets are also known as Hongbao, Red Envelopes, or Lucky Money. These Red Packets are based on the Chinese tradition of “Hongbao”, which are given by elders or friends as gifts. The money was directly deposited into a user’s WeChat Pay account or related bank account. WeChat Pay is similar to PayPal or Apple Pay and can be applied in online or real-life shopping.

In this study, we set up inclusion and exclusion criteria, and the specific procedures we used to check participants’ eligibility are described as follows:

(a) Participants contacted and “friended” the interventionist on WeChat.
(b) The interventionist asked participants the following questions:
  - In your entire lifetime, have you smoked at least 100 cigarettes? (If yes, go to the next question; if no, not eligible)
  - Do you currently smoke cigarettes? (If yes, go to the next question; if no, not eligible)
  - Do you currently live in China? (If yes, go to the next question; if no, not eligible)
  - Are you 18 or older? (If yes, go to the next question; if no, not eligible)
  - Do you use WeChat at least once a day? (If yes, go to the next question; if no, not eligible)
  - Our study is about smoking cessation. We will provide you videos, slides, pictures, texts, and social support to help you quit smoking. The duration of our study is about 3 weeks. Would you like to join our study? (If yes, eligible; if no, not eligible)

If all the answers were “yes”, the participants were eligible and consented to join the study. Eligible participants were randomly assigned to either intervention group 1, 2 or a control group. The first eligible participant was placed in intervention group 1; the second eligible participant was placed in intervention group 2; the third eligible participant was placed in the control group; the fourth eligible participant was placed in intervention group 1, and so on. Then, participants were required to provide their last 4 digits of their most frequently used phone number as a smoker ID, which was used to rename their screen name.

Participants who were not eligible could still “friend” the intervention account and were able to review the project WeChat Moments but were not able to interact with eligible participants or participate.

### 2.3. Intervention Content Categories 

One construct of the TTM is the “stage of change”, which consists of 5 stages. We adapted 4 stages for this study: Pre-contemplation, Contemplation, Preparation, and Action [16]. A total of 10 constructs of processes of change of the TTM are associated with specific stages and are used to guide interventions [16]. Our intervention consisted of 6 concepts.

- Consciousness Raising: The aim of this concept is to help participants understand the risk of smoking. According to the WHO, studies have shown that few people in developing countries understand the specific health risks of tobacco use [22]. For example, a 2009 survey in China revealed that only 38% of smokers knew that smoking caused coronary heart disease, and only 27% knew that it also caused strokes [22]. Six messages underlined this concept:
  o Message 1: Harms of smoking.
  o Message 2: Benefits of quitting smoking.
  o Message 3: Reasons to quit.
  o Message 4: What is stopping you from quitting smoking?
  o Message 5: Understanding nicotine.
  o Message 6: Smoking cessation medications.
- Self-efficacy: According to Bandura, self-efficacy is the most important personal factor in behavior change [16]. Self-efficacy has been applied to smoking cessation for several decades, and many studies have proven its effectiveness in smoking cessation [17]. Four messages highlighted this concept:
  o Message 7: Success stories from famous movie star Jackie Chan.
  o Message 8: Success stories from Taiwan celebrity Peiwei Ni.
  o Message 9: How to make a quit plan.
  o Message 10: Self-management approaches.
- Helping relationships: “Helping relationships” stems from seeking social support for healthy behavior change [16]. Social support includes both verbal and nonverbal communication, and usually refers to physical and emotional comfort given to us by our family, friends, co-workers, and others in our social networks [16]. Two messages emphasized this concept:
  o Message 11: Setting up the social support you need.
  o Message 12: Seeking social support from us.
- Stimulus control: Stimulus control refers to removing reminders or cues for unhealthy behaviors and adding cues or reminders to engage in a corresponding healthy behavior [16]. Prior studies have shown that higher stimulus control is associated with lower cigarette intake in daily smokers [23]. Two messages underlined this concept:
  o Message 13: Identifying common triggers and techniques.
  o Message 14: Saying “no” to people who hand you cigarettes.
- Coping skills: These are methods that people use to deal with stressful situations [16]. The process of smoking cessation is often difficult. Obtaining and maintaining good coping skills can lead to better cessation outcomes. Six messages addressed this concept:
  o Message 15: Coping with withdrawal symptoms.
  o Message 16: Preventing slips and relapse.
  o Message 17: Physical relaxation techniques.
  o Message 18: Mental relaxation techniques.
  o Message 19: Weight management through exercise.
  o Message 20: Weight management through diet.
- Oral Health Awareness: Oral disease is a major public health problem, with many oral diseases highly prevalent worldwide, especially in developing countries [13]. China has a high prevalence of many oral diseases; however, the severity of many oral diseases is often underestimated. Six messages addressed this concept:
  o Message 21: Smoking and oral health.
  o Message 22: Oral health problems.
  o Message 23: How to take care of teeth.
  o Message 24: Benefits of receiving routine teeth cleaning and dental checks.
  o Message 25: The necessity of ultrasonic teeth cleaning.
  o Message 26: Common questions about teeth cleaning.

### 2.4. Intervention Procedures

We developed 26 intervention messages (18 video-based smoking cessation messages, 2 picture-based smoking cessation messages, and 6 video-based oral health related messages) based on the transtheoretical model (TTM) and materials from the Louisiana Tobacco Control Initiative. The message types included imagery, text, and videos. We delivered intervention messages once a day during weekdays 8:00 a.m.–9:00 a.m. (China Standard Time, GMT +8) via WeChat broadcasting messages, with two messages being sent each time. The interventionist was able to send intervention messages to multiple participants at the same time (up to 200 at a time), according to WeChat version 7.0.3. Intervention messages were also posted on the Chinese Clinical Nutrition Network, a WeChat public account. These messages were forwarded to the project’s WeChat Moments, but the participants in the waitlist control group were excluded from viewing those intervention messages during the intervention period.

Every Friday night (China Standard Time, GMT +8) a group discussion was facilitated by the interventionist. A discussion for groups 1 and 2 was facilitated between 9:00 and 9:30 pm. The group discussion was related to the content that was delivered during that week, and the duration was around 30 min. Participants were able to ask any topic-related questions, and an interventionist answered these questions immediately. The questions and responses from smokers and interventionists were summarized and posted on WeChat Moments. Group members were able to mute notifications if they did not wish to participate and could search the chat history to view previous chatting records. Before the post-intervention period, participants could also ask the interventionist any smoking-related questions, and the interventionist responded to the question within 24 h.

### 2.5. Evaluation and Analyses

The name of participants was shown as their unique Smoker ID, which was also used to link their surveys. After the discussion, a process evaluation assessment was conducted, which consisted of a questionnaire survey and participant count. The measurements of the WeChat-based smoking cessation intervention study included process evaluation and outcome measurements. In this article, we focus on the process assessments. The process assessments were conducted every Friday to assess intervention message receipt, satisfaction level, engagement level, and likelihood of recommendation to others. The intervention message was measured by a self-reported question administered at each process evaluation assessment: “Please check the boxes if you have read the intervention messages this week.” Satisfaction assessments included intervention messages, group discussions, timing, questions and answers, and interventionists. Satisfaction was measured by 5 indicators: (1) “How satisfied were you with the intervention messages overall?”; (2) “How satisfied were you with group discussions?”; (3) “How satisfied were you with the time scheduling for group discussions?”; (4) “How satisfied were you with the Q&A session?”; and (5) “How satisfied were you with the interventionist?” Satisfaction was measured by a five-item Likert scale survey. Responses ranged from 1 = “very dissatisfied” to 5 = “very satisfied”. If the total score was greater than 20, then we considered the participants to be satisfied with the overall project. Intervention message engagement was measured by a self-reported question at each process evaluation: “This week, how engaged were you in the intervention messages?” Response options included highly engaged, somewhat engaged, neutral, not really engaged, and not engaged at all. Recommendation to others was measured using the following question: “Would you recommend our program to others?” Response options included “yes” and “no”.

Descriptive statistics were used to compare the baseline participants’ characteristics between the 3 intervention groups, as well as between the participants who completed surveys and those who did not complete the survey. Then, we tested whether there were significant differences. Chi-square/Fisher’s exact tests were used for comparison among categorical variables, and ANOVA was used for continuous variables. Two-tailed tests were performed with the significance level at 0.05. Results for the study population were based on their baseline assessments. Results for messages read during intervention weeks, satisfaction level, engagement level, and likelihood of recommendation to others were based on the actual number of participants who submitted process surveys. No significant differences were found between the participants who completed surveys and those who did not complete the survey for most of demographic indicators, except for sex. Males were more likely to complete assessments immediately after the intervention than females (92.6% vs. 83.9%).

## 3. Results

### 3.1. Study Population

Participants were recruited from 1 July to 5 August 2019, using the WeChat platform. Of the 1132 people who “friended” our project on WeChat, 729 were excluded due to a failed eligibility check, non-consent for participation, or an incomplete/invalid baseline survey. Thus, 403 people were eligible, consented to participate in the study, completed the baseline assessment, and were randomly assigned to intervention group 1, group 2, or the control group (group 3). As a result, 136, 135, and 132 smokers were randomly assigned to group 1, 2, and 3, respectively. At week 1, after the first week of smoking-related information delivery, 70 and 72 participants submitted process evaluation surveys from group 1 and group 2, respectively. At week 2, after completion of all smoking cessation related messages, 74 and 70 participants submitted process evaluation surveys from group 1 and group 2, respectively. At week 3, after the additional week of oral health related program content, 80 participants from group 2 submitted a survey.

Table 1 shows the detailed demographic information at baseline for each group. Descriptive statistics were used to describe demographic and overall health status characteristics for the study sample. Characteristics included age, gender, education level, income level, household registration, self-reported living areas, occupation, BMI, age at smoking initiation, and initial TTM stage of change. No significant differences were found between groups when examining all demographic indicators. The mean and standard deviation (SD) for participants’ overall age was 30.5 (9.6) and the mean (SD) of age at smoking initiation for all participants was 18.1 (4.1). The majority of participants (93.9%, *n* = 260) were less than 40 years old; 88.8% (*n* = 358) of the participants were male; 36.0% (*n* = 145) of participants had a household income under ¥50,000 ($7200); 29.1% (*n* = 122) of participants earned between ¥50,000–99,999 ($7200–15,000). According to the Chinese government, the poverty line for one person, per year, in 2018 was ¥3700 ($530)—a circumstance which characterizes about 10% of the total Chinese population. Most participants (57.8%, *n* = 233) were living in urban areas. Approximately four in ten participants had a high school education or less. About six in ten participants were married, and about four in ten were single. A total of 44.9% (*n* = 181) of participants worked in business. Approximately six in ten participants were underweight or normal weight.

### 3.2. Messages Read during the Intervention

Table 2 describes the messages given to participants during the intervention. Participants received two intervention messages from the interventionist, per day, from Monday to Friday during week 1. Participants were included if they completed process evaluation assessments and read at least one message during the intervention. No statistically significant differences were found in the number of messages read between group 1 and group 2. Participants reported reading at least one intervention message at week 1, week 2 (completion of smoking related messages), and week 3 (the additional week of oral health related program content). Participants read an average of 4.76 (out of 10), 5.80 (out of 10), and 4.25 (out of 6) messages at week 1, week 2, and week 3, respectively. For most of intervention days, the second messages (52.7%) were less likely to be read compared to the first messages (62.5%) for group 2 participants. This trend was also found for group 1 participants (48.1% for the second message vs. 56.0% for the first message).

**Figure 2:**
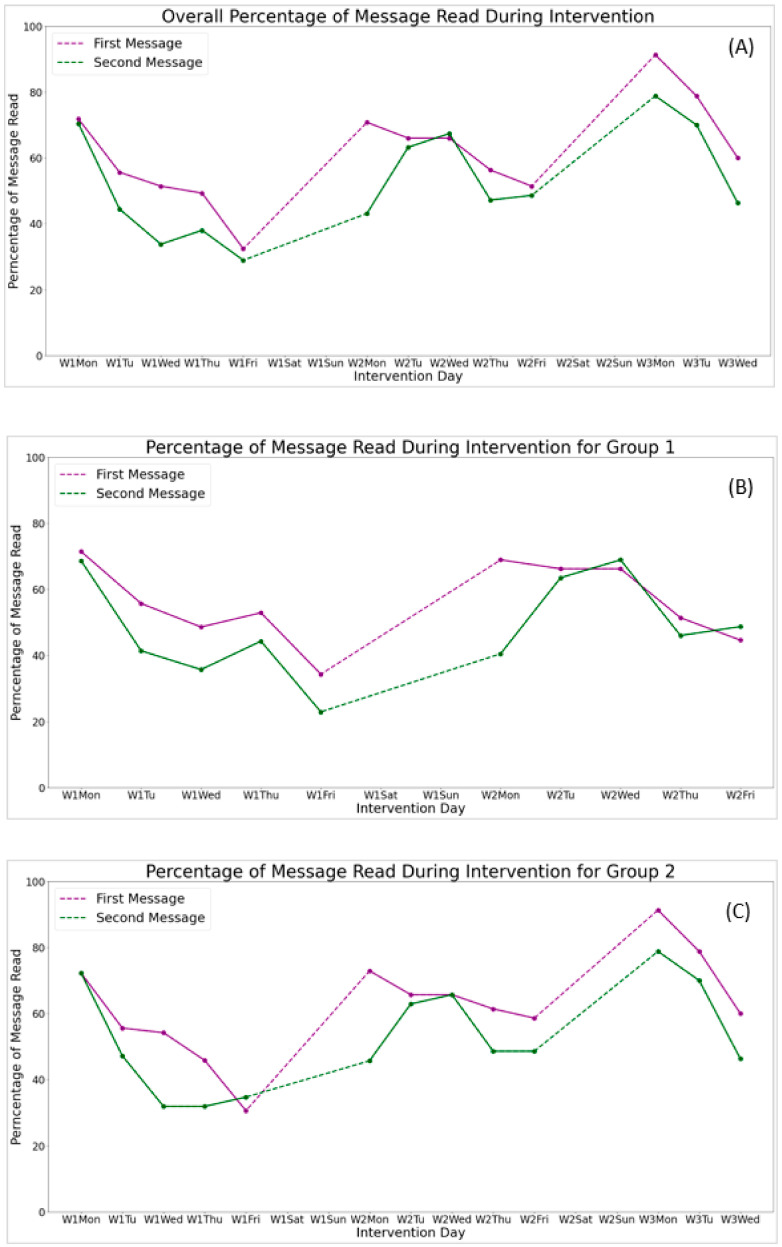
Percentage of messages read by participants during the intervention. (**A**): Overall percentage of message read during intervention; (**B**): Percentage of message read during intervention for Group 1; (**C**): Percentage of message read during intervention for Group 2.

Figure 2A–C describe the percentage of participants who read messages between groups, respectively. For each week, as the week went on, fewer and fewer participants read intervention messages; moreover, within each single week, the number of participants who read the intervention messages gradually decreases over time. However, participants had a higher rate of reading intervention messages during the beginning part of each week. Picture-based intervention messages were less likely to be read than video-based intervention messages.

### 3.3. Satisfaction Scores for Intervention

Table 3 describes satisfaction levels for the intervention. Total satisfaction scores ranged between 5 and 25, and the overall scores for satisfaction for each week were 21.55, 22.27, and 22.76, respectively. No significant differences were found for all satisfaction indicators between group 1 and group 2 at intervention week 1 and 2 (week 3 only included group 2, as this is the additional week for oral health-related information). About 40–70% of participants reported being very satisfied for each indicator across each week of the intervention. At week 1, after the first week of smoking related information delivery, 46.5% of participants reported being very satisfied with the intervention messages overall; 42.3% of participants reported being very satisfied with the content of group discussions; 43.0% of participants reported being very satisfied with the time scheduling for group discussions; 59.2% of participants reported being very satisfied with the Q&A session; 53.5% reported being very satisfied with the interventionist. The percentages of participants who reported being very satisfied at week 2 for each indicator were higher than those reported at week 1. At week 2, after completion of all smoking cessation related messages, 58.3% of participants reported being very satisfied with the intervention messages overall; 56.3% of participants reported being very satisfied with the content of group discussions; 47.9% of participants reported being very satisfied with the time scheduling for group discussions; 66.7% of participants reported being very satisfied with the Q&A session; 58.3% reported being very satisfied with the interventionist. Similarly, the percentages of participants who reported being very satisfied at week 3 for each indicator were higher than those reported at week 2. At week 3, after the additional week of oral health-related program content, 70.0% of participants reported being very satisfied with the intervention messages overall; 60.0% of participants reported being very satisfied with the content of group discussions; 60.0% of participants reported being very satisfied with the time scheduling for group discussions; 70.0% of participants reported being very satisfied with the Q&A session; 58.7% reported being very satisfied with the interventionist.

### 3.4. Engagement Levels

Table 4 describes the engagement levels for the intervention by individual week. Most participants reported being either highly engaged (30.3%) or somewhat engaged (38.7%) at week 1. Similarly, 42.4% and 32.6% of participants reported being either highly engaged or somewhat engaged at week 2 (completion of all smoking cessation related messages), respectively. In week 3 (the additional week of oral health-related program content), the percentages of participants in group 2 who reported being highly engaged or somewhat engaged were 38.8% and 35.0%, respectively. No significant differences were found in engagement levels between group 1 and group 2 for both week 1 and week 2.

### 3.5. Recommendation to Others

Table 4 describes participants’ likelihood of recommending the program to others by week. Most participants (93.0% at week 1, 95.8% at week 2, and 96.2% at week 3) reported that they were willing to recommend our program to others. A marginal significant difference in recommendation to others was shown between groups 1 and 2 at week 1, after the first week of smoking related information delivery. Participants in group 2 (97.2%) were more likely to recommend our program to others compared with those in group 1 (88.6%), *p* = 0.05. However, no significant differences were found in the likelihood to recommend the program to others between group 1 and group 2 at week 2, after completion of all smoking cessation related messages.

## 4. Discussion

In this implementational study, we developed a novel, low-cost, social media-based smoking cessation intervention. Our study found that smokers had a higher rate of reading intervention messages at the beginning part of each week; in addition, using videos to develop intervention messages might help to engage future participants. No statistically significant differences were found between group 1 and group 2 in terms of the number of messages read, satisfaction scores for intervention, engagement levels, and likelihood of recommendation to others. Below, we discuss the implementation of a WeChat-based smoking cessation study.

### 4.1. Messages Read during Intervention

Our study found that the order of delivery and the types of intervention messages had impact on the messages read during the intervention. For most of the intervention days, the second messages were less likely to be read compared with the first ones. Thus, for future interventions, one should consider sending messages of a higher importance as the first message of a day. Moreover, within each single week, the number of participants who read the intervention messages gradually decreases over time. This trend was in line with previous findings [24]. It would be beneficial for future researchers to investigate potential ways to maintain the reading rate across the week. In addition, we observed a higher reading rate at the beginning part of each week. This finding was consistent with the study by Cavallo et al., which enrolled 55 participants for a 12 week weight loss intervention using Facebook [25]. Cavallo et al. reported that the participants contributed more at the beginning part of the week in their text-based intervention study [25]. As a result, future interventions should consider sending the relatively more important messages at the beginning part of the week.

It is also worth noticing that the percentage of messages read on the first day of week 3 was boosted to 91.3%, compared with a 71.8% maximum over the previous two weeks. This was caused by the fact that we shifted to oral health-related messages, after delivering smoking cessation related messages for two weeks, so that the contents of the intervention messages are attractive and fresh to the participants. In addition, in our intervention, we delivered 18 smoking cessation-related video messages, 2 smoking cessation-related picture messages, and 6 oral health related-video messages. We found that picture-based intervention messages were less likely to be read than video-based intervention messages. This result was also in line with previous findings [25]. Study indicated that video messages produce, on average, more reactions than picture messages [25]. For future studies, one might consider alternating the topics and formats of the messages for better engagement from the users.

### 4.2. Strengths and Limitations

This study was an important step in exploring and examining the implementation of WeChat-based smoking cessation intervention. The study results can help to improve WeChat-based intervention for increased satisfaction and engagement, and most people were willing to recommend the study to others. To our knowledge, few studies have tried to used WeChat for smoking cessation intervention. Although we only tested a small group population, our study shows that the use of WeChat provides a promising and low-cost strategy for smoking cessation interventions. This is the first implementation study that used WeChat for smoking cessation intervention and for conducting assessments.

Although data from this evaluation shows that WeChat can be used for smoking cessation, it is important to note some limitations. First, the measurements of this study relied on self-reported data; thus, some recall and social desirability bias might exist among respondents. For example, participants’ emotions and environment at the time they filled out the questionnaire may be dominant factors. Second, this is a single-blind study. Despite participants being unaware of their own group placement, the interventionist knew the group placements. As a result, the interventionist may have inadvertently paid more attention to those participants who were in intervention groups; consequently, impacting the smoking cessation outcomes. In addition, participants were encouraged to forward advertisements on WeChat Moments and other social media platforms in a “word-of-mouth” recruitment approach; thus, it is difficult to estimate the total number of people that were reached out to.

### 4.3. Implications for Public Health

WeChat has over 1 billion users, including many middle-aged and senior users, as well as users who live in rural areas. Participants can participate in the intervention in simple ways, such as scanning a QR code from an advertisement. Moreover, WeChat can offer 24 h access to messages and live chats, which are features that other smoking cessation strategies—such as in-person behavioral counseling—are unable to provide. Furthermore, the cost of implementing a WeChat-based study is relatively low. In using WeChat for this intervention, the account can be set up as either an individual or an organizational account, depending on the implementing team’s goals. This is similar to other social media platforms (such as Twitter, Facebook, Instagram, or Clubhouse) making this intervention easily translatable to other platforms.

According to WHO reports, the need for smoking reduction and cessation is more urgent in developing countries like China than anywhere else in the world [22]. Moreover, China has one of the highest smoking rates and prevalence of oral disease cases in the world, and in China, oral health is often underestimated as a contributor to an individual’s overall health [9]. In 2016, the Chinese government released the Healthy China 2030 blueprint [26]. One central idea of these policies is to prevent oral diseases [26]. Therefore, our study’s aim to promote oral health awareness, in order to improve smoking cessation outcomes, is consistent with the government’s health plan. This harmony with governmental goals may contribute to success of wider implementation in the future.

## 5. Conclusions

We have implemented a WeChat-based smoking cessation intervention for Chinese smokers. For most of the intervention days, the second messages were less likely to be read compared to the first messages. Smokers had a higher rate of reading intervention messages during the beginning of each week, during which one should consider sending the relatively more important messages. Picture-based intervention messages tended to be less likely to be read than video-based intervention messages. One might consider alternating the topics and formats of the messages for a better engagement of the users for future studies. In addition, most of participants reported being satisfied, engaged, and willing to recommend our program to others.

## Figures and Tables

**Table 1:** Demographic information at baseline.

Variables		Group 1 (*n* = 136)		Group 2 (*n* = 135)		Group 3 (*n* = 132)		*p*-Value
		*n*	%	*n*	%	*n*	%	
Age (Mean, SD)		29.9	9.0	31.4	9.6	30.2	10.2	0.42
Age Category								0.64
	18–24	42	32.1	34	25.6	45	35.2	
	25–29	37	28.2	35	26.3	28	21.9	
	30–39	33	25.2	41	30.8	34	26.6	
	≥40	19	14.5	23	17.3	21	16.4	
Sex								0.23
	Male	117	86.0	119	88.2	122	92.4	
	Female	19	14.0	16	11.8	10	7.6	
Household Income ^1^ in ¥								0.30
	≤49,999	58	42.7	40	29.6	47	35.6	
	50,000–99,999	31	22.8	43	31.9	42	31.8	
	100,000–199,999	31	22.8	37	27.4	27	20.5	
	≥200,000	16	11.8	15	11.1	16	12.1	
Self-Reported Living Area ^2^								0.93
	Urban	79	58.1	80	59.3	74	56.1	
	Suburban	38	27.9	33	24.4	36	27.3	
	Rural	19	14.0	22	16.3	22	16.7	
Education Level								0.87
	High School or Less	51	37.5	55	40.7	47	35.6	
	Associated College	44	32.4	39	29.0	46	34.9	
	College and Above	41	30.2	41	30.4	39	29.6	
Marital Status								0.73
	Married	78	57.4	83	61.5	81	61.4	
	Single ^3^	58	42.7	52	38.5	51	38.6	
Occupation								0.23
	Business ^4^	70	51.5	62	45.9	49	37.1	
	Government/Agency Officers/Professional Staff ^5^	24	17.7	22	16.3	29	22.0	
	Labor Workers ^6^	19	14.0	16	11.9	19	14.4	
	Self-Employed and Other ^7^	23	16.9	35	25.9	35	26.5	
BMI ^8^								0.58
	Underweight and Normal Weight	81	60.0	73	54.1	77	58.8	
	Overweight and Obese	54	40.0	62	45.9	54	41.2	
Age at Smoking Initiation (Mean, SD)		18.0	3.6	18.3	4.6	17.9	4.0	0.76

^1^ The exchange rate for USD to RMB is as follows: ¥6.5 = $1; ≤¥49,999 = ≤$7692; ¥50,000–99,999 = $7692–15,384; ¥100,000–199,999 = $15,384–30,769; ≥¥200,000 = ≥$30,769. ^2^ “Urban” includes people who are living in prefecture-level cities or county-level cities; “Suburban” includes people who are living in the areas beyond a city’s border; “Exurban” includes people who are living in towns; and “Rural” includes people who are living in villages. ^3^ “Single” includes never married, widowed, divorced, or living with partner. ^4^ “Business” includes managers, general office staff, and business service workers (e.g., salesmen, shop clerks, waiters, etc.). ^5^ “Professional Staff” includes doctors, teachers, lawyers, journalists, etc. ^6^ “Labor Workers” includes factory workers, and farmers/foresters/fishermen. ^7^ “Self-Employed and Other” includes self-employed, freelancers, retired, unemployed, students, and others. ^8^ Asian BMI standards are as follows: Underweight and Normal Weight—BMI ≤ 22.9; Overweight and Obese—BMI ≥ 23.

**Table 2:** Messages read during intervention weeks.

Messages	Order	Total		Group 1		Group 2	
		*n*	%	*n*	%	*n*	%
Harms of smoking (Week 1 day 1)	1	102	71.8	50	71.4	52	72.2
Benefits of quitting smoking (Week 1 day 1)	2	100	70.4	48	68.6	52	72.2
Reasons to quit (Week 1 day 2)	1	79	55.6	39	55.7	40	55.6
What is stopping you from quitting smoking? (Week 1 day 2)	2	63	44.4	29	41.4	34	47.2
Understanding nicotine (Week 1 day 3)	1	73	51.4	34	48.6	39	54.2
Smoking cessation medications (Week 1 day 3)	2	48	33.8	25	35.7	23	31.9
Success stories from Jackie Chan (Week 1 day 4)	1	70	49.3	37	52.9	33	45.8
Success stories from Peiwei Ni (Week 1 day 4)	2	54	38.0	31	44.3	23	31.9
Making a quit plan (Week 1 day 5)	1	46	32.4	24	34.3	22	30.6
Self-Management approaches (Week 1 day 5)	2	41	28.9	16	22.9	25	34.7
**Average “Yes” Percentage at week 1**			**47.6**		**47.6**		**47.6**
Setting up the social support you need (Week 2 day 1)	1	102	70.8	51	68.9	51	72.9
Social support from us (Week 2 day 1) §	2	62	43.1	30	40.5	32	45.7
Identifying common triggers and techniques (Week 2 day 2)	1	95	66.0	49	66.2	46	65.7
Saying no to people who hand you cigarettes (Week 2 day 2) §	2	91	63.2	47	63.5	44	62.9
Coping with withdrawal symptoms (Week 2 day 3)	1	95	66.0	49	66.2	46	65.7
Prevention slips and relapse (Week 2 day 3)	2	97	67.4	51	68.9	46	65.7
Physical relaxation techniques (Week 2 day 4)	1	81	56.3	38	51.4	43	61.4
Mental relaxation techniques (Week 2 day 4)	2	68	47.2	34	46.0	34	48.6
Weight management with diet (Week 2 day 5)	1	74	51.4	33	44.6	41	58.6
Weight management with exercise (Week 2 day 5)	2	70	48.6	36	48.7	34	48.6
**Average “Yes” Percentage at week 2**			**58.0**		**56.5**		**59.6**
Smoking and oral health (Week 3 day 1)	1	73	91.3			73	91.3
Oral health programs (Week 3 day 1)	2	63	78.8			63	78.8
How to take care of teeth at home (Week 3 day 2)	1	63	78.8			63	78.8
Benefits of receiving routine teeth cleaning and dental checks (Week 3 day 2)	2	56	70.0			56	70.0
The necessity of ultrasonic teeth cleaning (Week 3 day 3)	1	48	60.0			48	60.0
Common questions about teeth cleaning (Week 3 day 3)	2	37	46.3			37	46.3
**Average “Yes” Percentage at week 3**			**70.9**				**70.9**
Summary the first message			61.6		56.0		62.5
Summary the second message			52.3		48.1		52.7

The total number of people who submitted this report at week 1 was 142 (group 1: 70, group 2: 72); the total number of people who submitted this report at week 2 was 144 (group 1: 74, group 2: 70); the total number of people who submitted this report at week 3 was 80. §—picture-based intervention message.

**Table 3:** Satisfaction scores for the intervention.

Variables	Total		Group 1		Group 2		*p*-Value
	*n* (Mean)	% (SD)	*n* (Mean)	% (SD)	*n* (Mean)	% (SD)	
Intervention messages overall at Week 1 (Range 1–5)	4.30	0.81	4.32	0.79	4.26	0.82	0.63
Content of group discussion at Week 1 (Range 1–5)	4.19	0.82	4.23	0.78	4.15	0.85	0.58
Time scheduling for group discussion at Week 1 (Range 1–5)	4.18	0.82	4.11	0.84	4.25	0.80	0.33
Q&A session at Week 1 (Range 1–5)	4.48	0.69	4.44	0.71	4.51	0.67	0.54
Interventionist at Week 1 (Range 1–5)	4.40	0.73	4.39	0.75	4.42	0.71	0.80
Total satisfaction score per participant at Week 1 (Range 5–25)	21.55	3.18	21.50	3.16	21.60	3.21	0.57
Self-reported overall score for the program at Week 1 (Range 1–5)	4.59	0.69	4.57	0.63	4.61	0.74	0.73
Intervention messages overall at Week 2 (Range 1–5)	4.48	0.70	4.49	0.69	4.47	0.72	0.90
Content of group discussions at Week 2 (Range 1–5)	4.43	0.72	4.42	0.72	4.44	0.71	0.84
Time scheduling for group discussions at Week 2 (Range 1–5)	4.26	0.80	4.24	0.77	4.29	0.84	0.75
Q&A session at Week 2 (Range 1–5)	4.60	0.63	4.62	0.63	4.57	0.63	0.63
Interventionist at Week 2 (Range 1–5)	4.50	0.65	4.43	0.68	4.57	0.60	0.20
Total satisfaction score per participant at Week 2 (Range 5–25)	22.27	2.98	22.20	2.91	22.34	3.06	0.78
Self-reported overall score for the program at Week 2 (Range 1–5)	4.78	0.46	4.78	0.48	4.79	0.46	0.98
Intervention messages overall at Week 3 (Range 1–5)					4.60	0.69	
Content of group discussions at Week 3 (Range 1–5)					4.53	0.64	
Time scheduling for group discussions at Week 3 (Range 1–5)					4.49	0.69	
Q&A session at Week 3 (Range 1–5)					4.66	0.55	
Interventionist at Week 3 (Range 1–5)					4.48	0.76	
Total satisfaction score per participant at Week 3 (Range 5–25)					22.76	3.16	
Self-reported overall score for the program at Week 3 (Range 1–5)					4.78	0.48	

The range for satisfaction scores was between 1 and 5. Higher scores indicate higher satisfaction with the program. The total number of people who submitted this report at week 1 was 142 (Group 1: 70, Group 2: 72); the total number of people who submitted this report at week 2 was 144 (Group 1: 74, Group 2: 70); the total number of people who submitted this report at week 3 was 80.

**Table 4:** Engagement Levels and Recommendation to Others.

Variables	Total		Group 1		Group 2		*p*
	*n* (Mean)	% (SD)	*n* (Mean)	% (SD)	*n* (Mean)	% (SD)	
Engagement with the intervention at Week 1 (Range 1–5)	3.91	0.92	3.79	0.88	4.03	0.96	0.12
Would recommend our program to others at Week 1 (Yes)	132	93.0	62	88.6	70	97.2	0.05
Engagement with the intervention at Week 2 (Range 1–5)	4.16	0.87	4.15	0.77	4.14	0.96	0.97
Would recommend our program to others at Week 2 (Yes)	138	95.8	70	94.6	68	97.1	0.68
Engagement with the intervention at Week 3 (Range 1–5)					4.06	0.93	
Would recommend our program to others at Week 3 (Yes)					77	96.2	

Total number of people who submitted this report at week 1 was 142 (Group 1: 70, Group 2: 72); the total number of people who submitted this report at week 2 was 144 (Group 1: 74, Group 2: 70); the total number of people who submitted this report at week 3 was 80.

## Data Availability

The material that support this manuscript are available from the corresponding author (TL) upon reasonable request.
